# Primary porcine proximal tubular cells as an alternative to human primary renal cells *in vitro*: an initial characterization

**DOI:** 10.1186/1471-2121-14-55

**Published:** 2013-12-05

**Authors:** Alexandra H Heussner, Daniel R Dietrich

**Affiliations:** 1 Human and Environmental Toxicology, University of Konstanz, 78457 Konstanz, Germany

**Keywords:** Primary porcine proximal tubular cells, Transporter expression, OAT, MRP, MDR, OATP, Cellular uptake, *In vitro* model

## Abstract

**Background:**

A good *in vitro* model should approximate an *in vivo*-like behavior as closely as possible in order to reflect most likely the *in vivo* situation. Regarding renal physiology of different species, humans are more closely related to pigs than to rodents, therefore primary porcine kidney cells (PKC) and their subsequent cell strain could be a valid alternative to primary human cells for renal *in vitro* toxicology. For this PKC must display inherent characteristics (e.g. structural organization) and functions (e.g. transepithelial transport) as observed under *in vivo* conditions within the respective part of the kidney.

**Results:**

We carried out a comprehensive characterization of PKC and their subsequent cell strain, including morphology and growth as well as transporter expression and functionality. The data presented here demonstrate that PKC express various transporters including pMrp1 (abcc1), pMrp2 (abcc2), pOat1 (slc22a6) and pOat3 (slc22a8), whereas pMdr1 (abcb1) and pOatp1a2 (slco1a2) mRNA could not be detected in either the PKCs or in the porcine cortical tissue. Functionality of the transporters was demonstrated by determining the specific PAH transport kinetics.

**Conclusions:**

On the basis of the presented results it can be concluded that PKC and to some extent their subsequent cell strain represent a valuable model for *in vitro* toxicology, which might be used as an alternative to human primary cells.

## Background

Recent discussions on the value of *in vivo* rodent bioassays for risk assessment and risk extrapolation to humans demonstrate the growing uneasiness of using rodents as surrogates for humans. Indeed humans are not 70 kg rodents and thus it does not come as a surprise that rodents differ physiologically and anatomically dramatically from humans [1]. The ever growing number of well characterized species-and sex-specific mechanisms of toxicity and carcinogenicity in rodents that have no comparison to and thus no relevance for humans, e.g. the α2u-globulin nephropathy/carcinogenesis [2], sodium-glucose linked transporter (SGLT) inhibitor mediated renal carcinogenesis in mice or rats [3], D-amino acid oxidase (DAAO) droplet nephropathy in male and female rats [4] etc. are testimony of the problems associated with using *in vivo* rodent bioassays to understand mechanisms of toxicity and the extrapolation of potential risk to humans. Similarly, the use of rodent primary cells *in vitro*, albeit providing for a more defined and controlled testing system, is subject to the same restrictions with regard to the extrapolation of findings to the human situation. Consequently, there has been a recent emphasis on employing human primary cells as well as cell lines to allow delineation of the underlying mechanisms of toxicity. While human cell lines are derived from either cancer cells or have been transformed using viral genes or DNA alkylating or oxidizing reactions and thus bear little resemblance to ‘normal’ human cells, employing human primary cells would be the most optimal solution. However, human primary cells are difficult to obtain in sufficient amounts for obvious reasons and thus demand thinking of potential alternatives.

Indeed, the porcine kidney is anatomically and physiologically comparable with the human [5] and can be obtained without real restrictions and at relatively low costs. More importantly one of the real caveats in using cells *in vitro* is the variable expression of transporters, the type of transporters expressed, the level of expression, the homology/amino acid identity and tertiary and quaternary structures of the transporters as well as the transporter type distribution within a given anatomical subunit e.g. the proximal tubule epithelial cells at the basal membrane of the luminal surface. Indeed, of the many human transporters known, at least the fully sequenced porcine transporters have a higher structural/amino acid identity to the human homologues than those of either mice or rats (Additional file 1: Figure S1, Additional file 2: Table S1). The latter is also critical when kinetics as well as dynamics of given compounds that need active or passive transport across membranes are considered, as the higher the homology to the human transporter the higher the likelihood that transport affinity and capacity are similar in the human and porcine homologues for the compound. This assumption is supported by data available for OAT1 and to some extent also for OAT3, whereas for other transporters insufficient data is available for comparison (Additional file 3: Table S2). However, in order to employ primary porcine kidney cells (PKC) and their subsequent cell strain for compound assessment it is crucial to demonstrate the types and levels of transporters expressed, their functionality (transporting capability) as well as their consistent expression over several cell culture passages. In order to address the latter points of characterization, primary PKC were generated from kidneys obtained from German hybrid pigs and cultured over several days and passages. The latter resulted in a PKC cell strain with a finite lifespan according to the traditional definition [6,7]. Moreover, as transporters are expressed at the basolateral or luminal side of renal epithelial cells only, compounds for testing transport functionality were chosen that need two differently localized transporters for cellular uptake and excretion, respectively. Finally in order to determine whether the expression of given transporters are hormone or substrate dependent, specific treatments were employed to detect differences in expression transporter levels.

In addition, two continuous cell lines of rat (NRK-52E) and porcine (LLC-PK1) origin that are often used in *in vitro* renal toxicology were run alongside for comparison.

## Methods

### Materials

Unless stated otherwise, materials were purchased from the following commercial suppliers: PAA Laboratories GmbH, Cölbe, Germany (cell culture chemicals), Sarstedt, Nümbrecht, Germany (cell culture plastics), BD Biosciences, Heidelberg, Germany (Primaria™ cell culture plastic ware, filter inserts), Fermentas, St. Leon-Rot, Germany (molecular biology reagents), MWG Biotech AG, Ebersberg, Germany (PCR primers), Perkin Elmer, Rodgau-Jügesheim, Germany ([^3^H]MTX, 1.48 × 10^12^ Bq/mmol (40 Ci/mmol), [^14^C]PAH), 2.035 × 10^9^ Bq/mmol (55 mCi/mmol), Zinsser Analytic GmbH, Frankfurt, Germany (Quicksafe A scintillation cocktail for aqueous samples) and Sigma-Aldrich GmbH, Seelze, Germany (all other chemicals).

### Animal tissue and cell cultures

Whole kidneys from fresh-killed male improved German hybrid pigs were obtained from a local slaughter-house. Primary porcine kidney cells (PKC) were prepared by collagenase digestion from renal cortices and cultured as previously described in detail [8] with the difference that the Percoll step employed in the original method was omitted. Briefly, a piece of renal cortex was minced in ice-cold modified Hank’s buffered salt solution (5.36 mM KCl, 0.44 mM KH_2_PO_4_, 145 mM NaCl, 0.34 mM Na_2_HPO_4_, 10 mM HEPES, pH 7.4, 300 mOs/kg H_2_O) containing 0.2 mM EGTA and 200,000 U L^-1^ penicillin and 200 mg L^-1^ streptomycin sulfate (HBSS-EGTA). After washing in HBSS-EGTA, the tissue was digested with collagenase (Type I from *Clostridium histolyticum*) in HBSS (1 mg/ml) containing 4 mM CaCl_2_ and 1 mM MgCl_2_ at 37°C with gentle agitation for 20-30 minutes. Thereafter, the cell suspension was filtered through 200 μm and 40 μm gauze and washed twice with HBSS. The final cell pellet was resuspended in DMEM-D-Val (PAA, cat# E15-055), to suppress fibroblast growth, supplemented with 2 mM L-glutamine, 10% FBS (FBS gold, PAA, cat# A15-649) and antibiotics (100,000 U L^-1^ penicillin, 100 mg L^-1^ streptomycin sulfate) and seeded into Primaria™ plasticware. Cultures were maintained in a standard humidified atmosphere (95% O_2_, 5% CO_2_, 37°C). Medium was exchanged every second day and after one week, medium was replaced by DMEM/F12 medium (PAA, cat# E15-813) supplemented with 10% FBS and antibiotics. Cells were passaged using trypsin-EDTA and cells were then seeded onto standard cell culture plastics.

Cell viability was estimated using the Trypan Blue exclusion assay. Only preparations of ≥90% viability were used for culturing. Cell origin was determined by enzyme distribution, as described earlier in detail [8-10], using γ-glutamyl-transferase and hexokinase activity as markers for proximal and distal tubular origin, respectively. Only cell preparations of predominantly proximal tubular origin (>85%) were used for culture.

LLC-PK1 and NRK-52E cells were obtained from the European Collection of Cell Cultures, Salisbury, UK (ECACC #86121112) and from the DSMZ, Braunschweig, Germany (#ACC199), respectively. Cell lines were cultured in standard plastic ware with DMEM (PAA, cat# E15-806) containing 10% FBS and antibiotics (100,000 U L^-1^ penicillin, 100 mg L^-1^ streptomycin) under standard humidified conditions (95% O_2_, 5% CO_2_, 37°C).

Cell culture samples for RNA and protein extraction were prepared by standard trypsin-EDTA treatment followed by washing with PBS. Resulting cell pellets were snap-frozen and stored at -80°C until use. Renal tissue samples were obtained from a male Wistar rat (rKidney cortex) and from a male German hybrid pig (pKidney cortex), snap-frozen and stored at -80°C until use.

### Growth curves

Cells were seeded in 6-well plates at a density of 5 × 10^3^ cells cm^-2^ and medium was replaced every 48 hours. Three wells were counted (technical replicates) daily from day 1-10 (30 wells) using a Z1 particle counter (Beckman Coulter, Krefeld, Germany) after trypsin-EDTA treatment.

### Cytotoxicity experiments

Cytotoxicity experiments using the MTT reduction assay as previously described [8], were conducted to estimate non-toxic concentrations for subsequent analyses. All chemicals and stock solutions (Tables 1 and 2) were sterilized by filtration (0.2 μm) prior to testing. Briefly, after incubating the cells in the presence of MTT (250 μg mL^-1^) at 37°C for 1.5 hours, the supernatant was discarded and the intracellular formazan was solubilized with 95% (v/v) isopropanol/ 5% (v/v) formic acid. Absorbance was read at 550 nm using a microplate reader (Infinite M200, Tecan, Crailsheim, Germany).

**Table 1:** Summary of cytotoxicity data of transport substrates and inhibitors (24 hours of exposure)

**Substances (Abbrev.)**	**Solvent**	**Concentration range tested (n concentrations)**	**Significant effects**
cimetidine (CIM)	H_2_O	1 μM-948 μM (7)	no
estrone sulfate (ES)	MeOH	1 μM-1 mM (7)	no
p-aminohippuric acid (PAH)	H_2_O	1 μM-1 mM (7)	no
methotrexate (MTX)	50% EtOH	1 μM-500 μM (6)	no
probenecid (PROB)	50% EtOH	1 μM-1 mM (7)	no
folic acid (FA)	0.1 M bicarbonate	1 μM-560 μM (6)	no
bromosulphophthalein (BSP)	H_2_O	1 μM-500 μM (6)	no
ochratoxin A (OTA)	EtOH	1 μM-1 mM (7)	no
ochratoxins B (OTB)	EtOH	1 μM-500 μM (6)	no

Data represent n = 3-4 independent experiments, conducted in duplicate. One-way ANOVA with Dunnett’s multiple comparison test (P < 0.05).

**Table 2:** Summary of cytotoxicity data of potential transporter inducers (24-96 hours of exposure)

**Substances (Abbrev.)**	**Solvent**	**Concentration range tested (n concentrations)**	**Significant effects**
bromosulphophthalein (BSP)	H_2_O	10 nM-20 μM (7)	no
p-aminohippuric acid (PAH)	H_2_O	10 nM-20 μM (7)	no
taurocholic acid (TCA)	EtOH	10 nM-20 μM (7)	no
triiodothyronine (T3)	PBS	100 pM-6.2 μM (7)	no
dehydroepi-androsteron sulfate (DHEAS)	H_2_O	10 nM-20 μM (7)	no
testosterone (TEST)	EtOH	10 nM-4 μM (7)	no
dexamethasone (DEX)	50% EtOH	100 pM-10 μM (6)	no

Data represent n = 3-4 independent experiments, conducted in duplicate. One-way ANOVA with Dunnett’s multiple comparison test (P < 0.05).

Cytotoxicity was determined in the kinetic experiments (short substrate exposure/ high concentrations, Table 1) after 24 hr substrate exposure, thereby ensuring that a substrate exposure of up to 30 min will not encompass substrate cytotoxicity. Conversely, in the transport induction experiments (prolonged substrate exposure/low concentrations, Table 2) cytotoxicity was determined after 24, 48, 72 and 96 hours of exposure. All assays were performed with the respective vehicle controls (Tables 1 and 2) and cadmium chloride as positive control.

### Transporter induction experiments

For transporter induction experiments, freshly isolated PKC cells were seeded (total of 2 × 10^4^ cells cm^-2^) on 6-well ThinCert PET tissue culture inserts in standard culture medium and medium was changed every 48 hours. Cell cultures formed a tight monolayer on day 7 of culture, which was confirmed microscopically before use. At this time point medium was changed again and incubations with potential transporter inducers were started by adding 50 nM or 1 μM of bromosulphophthalein (BSP), p-aminohippuric acid (PAH), taurocholic acid (TCA), triiodothyronine (T3), dehydroepiandrosteron sulfate (DHEAS), testosterone (TEST) and dexamethasone (DEX) to the basolateral compartment. After 48 hours of exposure, samples for RNA extraction were prepared by standard trypsin-EDTA treatment followed by washing with PBS. Resulting cell pellets were snap-frozen and stored at -80°C until use.

### RNA extraction and RT-PCR analysis

Total RNA was prepared from cell pellets and minced pieces of tissue (rat kidney and porcine kidney cortex) via the phenol-chloroform method using Trizol (Invitrogen, Karlsruhe, Germany). Concentration and quality was estimated via 260/280 nm values. Representative samples were tested for RNA integrity via denaturing electrophoresis. cDNA was prepared using 1 μg total RNA, Oligo (dT)18 and M-MuLV reverse transcriptase according to manufacturer’s instructions. PCR was performed using 2 μl cDNA sample, 2 × PCR Master Mix, specific primers (Table 3). Amplification was performed with an initial denaturation step at 92°C for 3 min followed by 30 cycles of 94°C for 60 sec, 58°C for 60 sec and 72°C for 60 sec and a final extension step at 72°C for 7 min.

**Table 3:** Primer sequences

**Name**	**Sequence (forward)**	**Sequence (reverse)**	**Product size (bp)**
pGAPDH	5′-ggg cat gaa cca tga gaa gt-3′	5′-agg cag gga tga tgt tct gg-3′	230
pOat1	5′-tac act ggg gag ctg tac cc-3′	5′-tct ttt cct cct gct ttc ca-3′	246
pOat3	5′-ctt cct gtt gtc ctg gtg gt-3′	5′-ggg aaa gac aga ggg tca ca-3′	248
pMdr1	5′-tcc agt ttc ctt ttg gag ga-3′	5′-tgt cct gtc gtt tgg ttt ca-3′	176
pMrp1	5′-gca gat cac cgc ata ctt ga-3′	5′-gtc cag gtc gtc tcg gta ac-3′	219
pMrp2	5′-gct tgc agt tcg tct gga gt-3′	5′-caa cag cca caa tgt tgg tc-3′	191
pOatp1a2	5′-atg ggc cta ggg tgt ttc tt-3′	5′-agg cat gat ggg agt ttc ac-3′	246

Primer design was performed on the basis of sequence data available at NCBI (http://www.ncbi.nlm.nih.gov) using Primer3 V0.2 software [47], which is online available (http://www.primer3.sourceforge.net) with default settings.

PCR products were separated on 1.5% agarose gels with 1 × TAE buffer and stained with ethidium bromide. A MultiDoc-It digital imaging system (UVP, LTF Labortechnik, Germany) was used for photographic documentation.

All nucleic acid analyses were performed with appropriate control samples in parallel (water samples, samples without addition of reverse transcriptase, etc.).

PCR products were sent to MWG, MWG-Biotech AG, Ebersberg, Germany) for automated sequence analysis.

### Protein preparation and analysis

Enriched membrane protein fractions were prepared according to Scalera *et al.* with modifications [11]. Briefly, cell pellets or minced tissue pieces were resuspended in sucrose buffer (250 mM sucrose, 10 mM Tris-HCl (pH 7.4), 1 mM EDTA, 1 mM PMSF) using a ratio of approximately 1:5 (w/v). Crude homogenates were prepared using a motor-driven teflon potter followed by an ultrasound step at 4°C. Cellular debris was removed by centrifugation (1,000 × g, 5 min, 4°C) and the supernatants were centrifuged again (6,000 × g, 5 min, 4°C). The pale fractions of the pellets were combined with the supernatants and centrifuged (16,000 × g, 30 min, 4°C). The resulting pellets (i.e. enriched membrane fractions) were resuspended in a minimal volume of sucrose buffer and total protein contents were determined using the Bradford assay according to manufacturer’s instructions (Biorad, Germany). Samples were diluted to a final concentration of 3 mg/ml in Laemmli sample buffer and incubated for 30 min at 37°C. Proteins were separated on a 12% polyacrylamide gel (20 μg protein/ lane) and electro-blotted onto a nitrocellulose membrane. After blocking (100 mM Tris-HCl pH 7.6, 0.9% NaCl, 0.1% Tween 20, 0.1% BSA), immune-detection was performed using rabbit anti-rat Oat1 and Oat3 (polyclonal, unpurified serum, Alpha Diagnostics, San Antonio, USA) and rabbit anti-human MRP1 and MRP2 (polyclonal, Abcam, Cambridge, UK) in a dilution of 1:5,000 and 1:1,000, respectively, in blocking buffer overnight, washing with TTBS (100 mM Tris-HCl pH 7.6, 0.9% NaCl, 0.1% Tween 20), followed by incubation with the secondary antibody (goat anti-rabbit IgG-HRP, 1:80,000) for 1 hr at room temperature. After additional washing, chemiluminescent detection was performed using ECL Plus and Hyperfilm ECL (GE Healthcare, Germany). The detection of pOAT1 and pOAT3 by specific antibodies was confirmed using the respective control blocking peptides (16 aa peptide of rat OAT 1 or OAT3 that was used as part of the antigen for antibody production) according to manufacturer’s instructions (Alpha Diagnostics, San Antonio, USA).

### Transport assays (kinetics)

For transport studies, PKC were seeded (1 × 10^5^ cells/cm^2^) on 6-well PET filter inserts with 0.4 μm pore size. The use of filter inserts allows measurement of transport across the BLM (basolateral membrane) and the BBM (brushborder membrane) by addition of substrates to media in the lower and upper compartment, respectively. After seven days of standard culture conditions a tight cell monolayer was present and culture medium was replaced with assay medium (without FBS), containing the appropriate concentration of substrate and 925 Bq (=25 nCi) of radiolabelled substrate. The cells were incubated at 37°C for the indicated times. After incubation, the media from the upper and lower chamber were saved and the filter inserts were washed carefully three times with ice-cold PBS (136.9 mM NaCl, 2.7 mM KCl, 8.1 mM Na_2_HPO_4_, 1.5 mM KH_2_PO_4_, pH 7.4) and cells were solubilized with 0.5 M NaOH for at least one hour at RT. Thereafter, cells were acidified with 1 M HCl and an aliquot was used for protein determination using the Bradford assay. 1 ml of medium from each compartment and of solubilized cells was placed into 10 ml scintillation cocktail and radioactivity was measured in a Beckman LS 6500 scintillation counter. In addition, several controls were employed (medium, solubilization solution, solubilized cells, total radioactivity (925 Bq) and total radioactivity (925 Bq) in the medium) to determine blanks, color quenching and handling variability.

Transport time courses were determined using a mixture of 50 μM substrate and 925 Bq radiolabelled substrate and 0, 1, 5, 10 and 30 min time points. As preliminary experiments demonstrated that no further increase of uptake could be obtained after 60 and 120 min incubation, these longer substrate exposure time points were omitted from further experimentation.

Transport concentration dependency was determined using nominally 0, 1, 5, 10, 20, 50 and 100 μM substrate, supplemented with radiolabelled substrate (925 Bq) and 30 min of incubation. The solutions used were measured in parallel to the experiment in order to determine the real concentrations applied resulting in 0.2, 1.1, 4.8, 9.5, 18.7, 46.3 and 92.2 μM. Inhibition studies were performed using a mixture of 50 μM substrate, 925 Bq radiolabelled substrate and inhibitors (1 mM) and 10 min incubation time.

For transport studies, tightness of the cellular monolayers was inspected microscopically before use. Additionally, radiolabelled substrates were measured in the both chambers (above and below the cell layer) to determine cell layer leakiness. In case of lacking or reduced tightness of the monolayer, very high counts (dpm) were measured in the respective opposite compartment. These resulting counts (dpm) were several-magnitudes greater than those that could have been expected from potential transepithelial transport. Data from cell preparations with such apparent cell layer leakiness were omitted from calculation.

### Data analysis

All numerical calculations and graphical representations were performed using MS Excel 2007 and GraphPad Prism 4.03. Data from growth curves were calculated as means ± SD from three technical replicates. Cell number doubling times were estimated from the logarithmic phase of the growth curve.

Cytotoxicity data were calculated as means ± SEM from at least three independent replicate experiments conducted in technical duplicates, followed by a One-way ANOVA with a subsequent Dunnett’s multiple comparison test (P < 0.05). For the positive control (CdCl_2_), a non-linear regression (5-PL, 95% CI) analysis was performed and effective concentrations (EC_50_) determined.

For kinetic data, raw transport data (dpm) were submitted to intra-assay normalization using the blanks described above and calculated using the following equation:

$$\begin{array}{c} \frac{\mathrm{mean}\;\mathrm{dpm}\times \mathrm{total}\;\mathrm{volume}}{\mathrm{counted}\;\mathrm{volume}\times 60\;\sec\times \mathrm{specific}\;\mathrm{activity}\times \mathrm{protein}\;\mathrm{content}\times \mathrm{assay}\;\mathrm{time}}\\ \;=\mathrm{substrate}\;\left(\mathrm{pmol}/\mathrm{mg}\times \min\right) \end{array}$$

Non-linear regression (5-PL, 95% CI) analysis was performed on data of 3-4 independent replicate experiments conducted in technical duplicates providing for a K_m_ and R^2^.

Densitometry was performed on agarose gels using Biorad QuantityOne V4.6.1. Each value was normalized with the respective GAPDH value. To reduce residual background noise from differing absolute values of independent replicate experiments, all experiments were run with the same positive control (RNA sample extracted from porcine kidney cortex), which were also used for intra-experimental normalization. Data were presented as mean transporter expression/ housekeeping gene expression vs. positive control/ housekeeping gene expression ± SEM from at least three independent replicate experiments.

## Results

### Basic characterization of PKC

**Figure 1:**
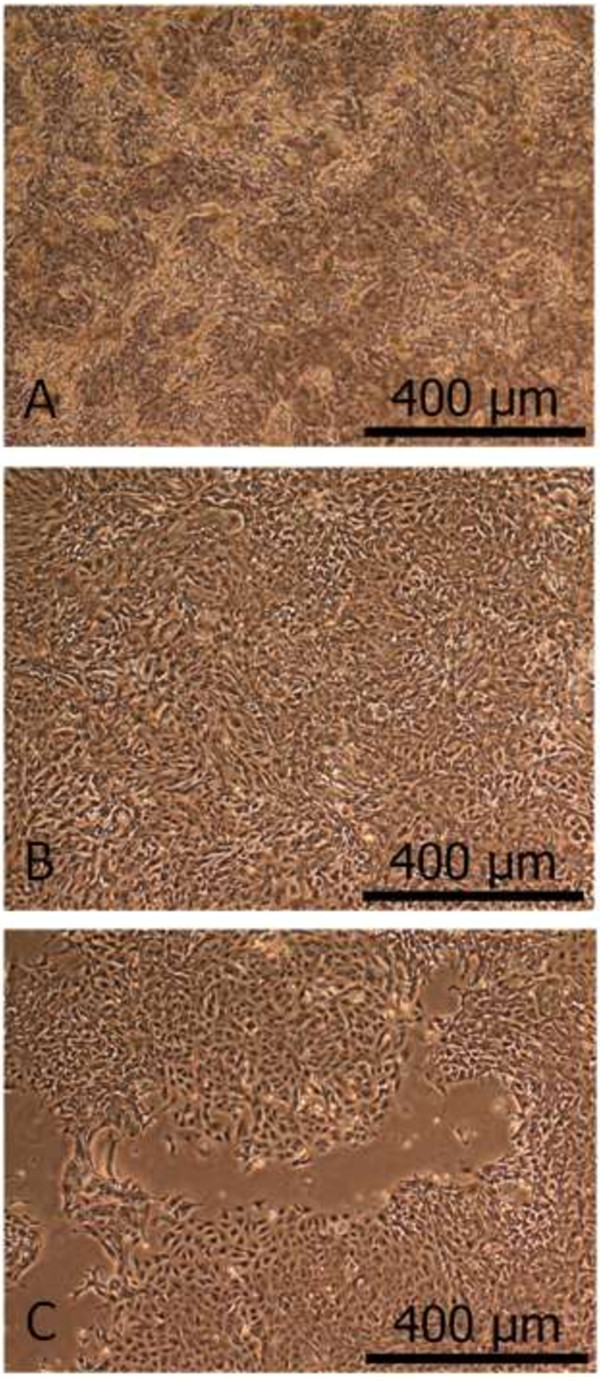
**Representative pictures of primary porcine kidney epithelial cells (PKC) at different states of confluence.** Pictures show PKC cultures of passage 0, 7 days after isolation with different confluence levels: **A**, post-confluent (>100% confluence); **B**, confluent (100% confluence); **C**, semi-confluent (~70% confluence); the used terms of confluence define the various density states of the cultures, i.e. non-confluent (described as <100% confluence), confluent (a state where the dish is completely covered with cells, described as 100% confluence) and post-confluent (a state, where the maximal cell number is reached, described as >100%). The latter does not mean that these cells grow in multilayers, but that they are able to close ranks more tightly before they stop proliferating due to contact inhibition.

**Figure 2:**
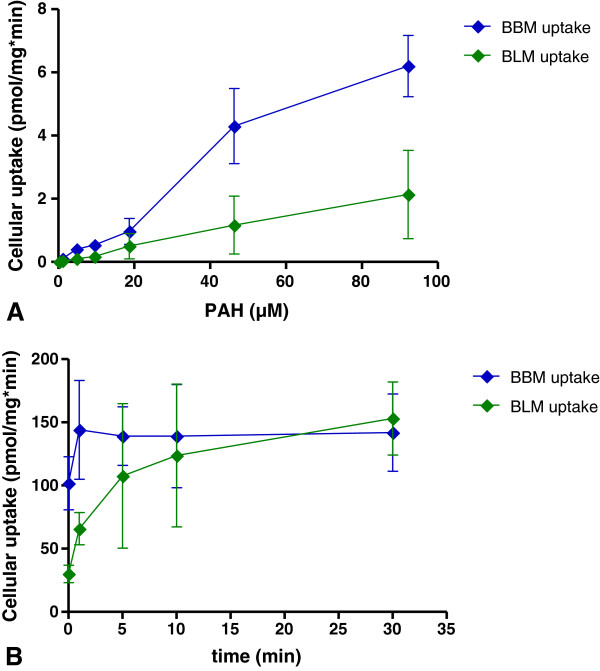
**Cellular PAH uptake. A**, uptake vs. PAH concentration; **B**, uptake vs. time; data represent means from 3-4 independent experiments performed in duplicates ± SEM.

**Figure 3:**
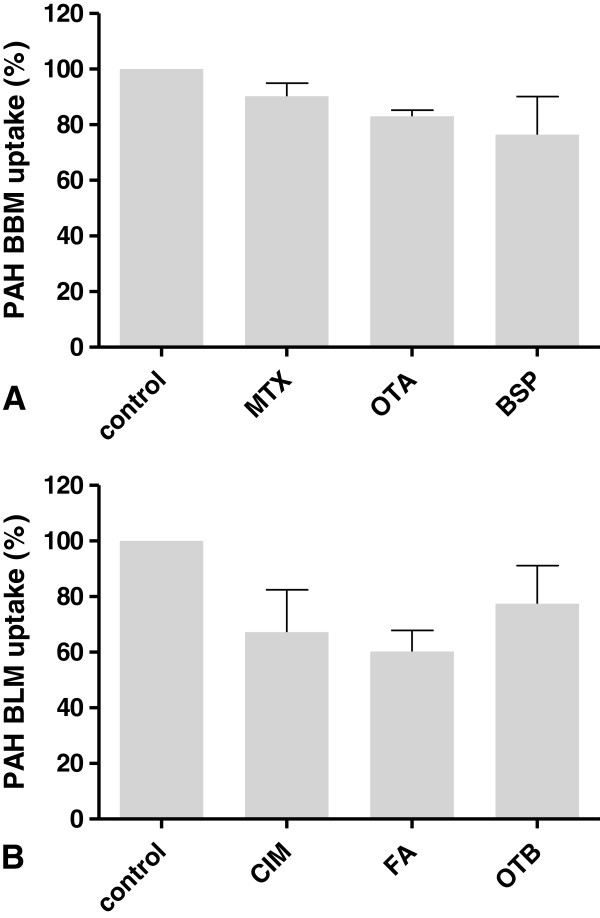
**Inhibition of cellular PAH uptake. A**, BBM (brushborder membrane) uptake; **B**, BLM (basolateral membrane) uptake; columns represent means from 3-4 independent experiments performed in duplicates ± SEM.

**Figure 4:**
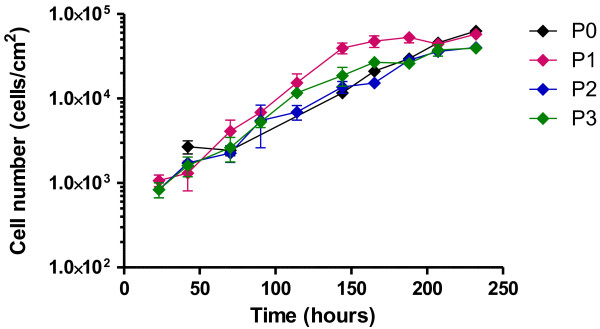
**Representative growth curves of PKC of passage 0-3.** Data represent means ± SD from 3 technical replicates.

**Figure 5:**
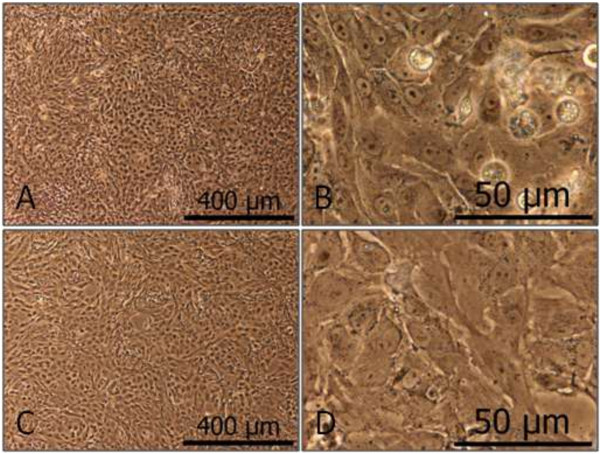
**Pictures of PKC at different passages.** Pictures show confluent PKC cultures of passage 0 **(A-B)** and 3 **(C-D)**.

PKC cultures formed monolayers with the typical epithelial appearance and domes (Figure 1). Additionally, a basolateral-apical polarity can be assumed at confluence on the basis of the transport experiments, as described below (Figures 2 and 3). PKC growth patterns were investigated several times in freshly isolated cell cultures as well as for subsequent cell passages. PKC showed a typical growth curve with a lag-phase of up to 50 hours, a maximum final cell density of almost 1 × 10^5^ cells cm^-2^ at confluence and a cellular doubling time of approximately 29 hours. No differences in cell number and cellular doubling time were usually observed between cell passage zero and three (Figure 4), however signs of degeneration or, more likely, senescence in individual cells (enlarged cell bodies with cytoskeletal changes (Figure 5D)) were apparent especially within cell layers of the otherwise normally looking passage three cell layer (Figure 5C).

### Expression of selected transporters at mRNA and protein level

**Figure 6:**
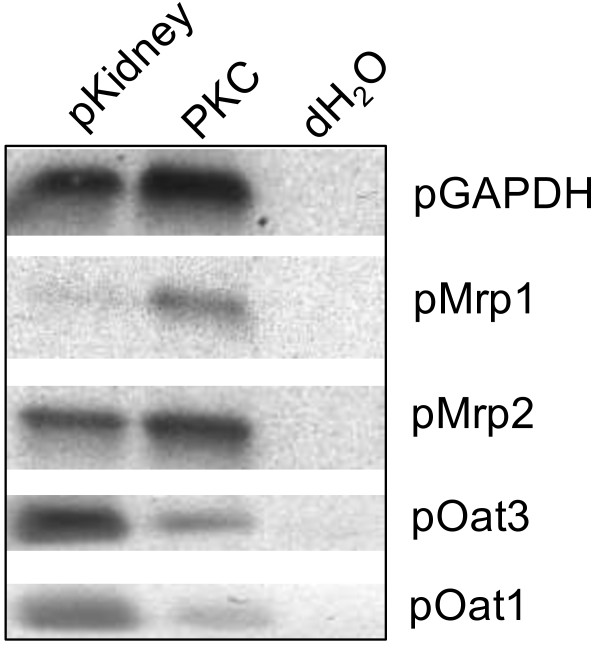
**Results of mRNA analysis (Representative picture).** 1.5% TAE-agarose gels with ethidium bromide UV-detection (picture colors are inverted); pKidney: RNA from porcine renal cortex; PKC, porcine kidney cells; dH_2_O, deionized water; pGAPDH: porcine glyceraldehyde phosphate dehydrogenase; pMrp1/2, porcine multidrug resistance-associated protein 1 and 2; pOat1/3, porcine organic anion transporters 1 and 3.

Gene expression analysis of transporter mRNA in PKC included pMdr1, pMrp1, pMrp2, pOatp1a2, pOat1 and pOat3. Porcine GAPDH (pGAPDH) was used as housekeeping gene and total RNA from porcine renal tissue (pKidney) and deionized water (dH_2_O) served as positive and negative controls, respectively. No signal could be obtained for pMdr1 and pOatp1a2 neither in the cells nor in the organ tissue preparations (data not shown). All other transporter mRNA was detectable in the tissue and cell samples (Figure 6) and the results were confirmed via sequencing of the respective RT-PCR products with 100% identity in each case. LLC-PK1 and NRK-52E cell lines were negative for all transporters tested (data not shown).

**Figure 7:**
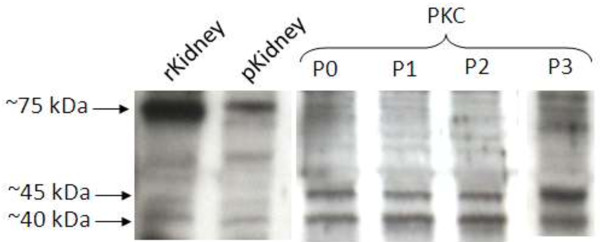
**Results of Oat1 protein analysis (Representative picture).** PKC, porcine kidney cells; rKidney, tissue from rat kidney; pKidney, tissue from porcine kidney; P0-3, passage number.

Protein analyses using SDS-PAGE followed by Western blotting and immunodetection with specific antibodies were performed only for transporters that were positively detected on mRNA level. For this purpose, enriched membrane fractions isolated from confluent PKC, NRK-52E and LLC-PK1 cultures and from porcine and rat renal cortex tissue were used. These experiments confirmed the mRNA expression data for pOat1 and pOat3 in PKC, although the protein bands were different from those obtained with the renal tissue samples from rat and porcine origin (Figure 7). The blots show immunoreactive proteins at approximately 75, 45 and 40 kDa and the specificity of the antigen-antibody reaction was demonstrated by preliminary experiments using the respective control peptides (Alpha Diagnostics) according to manufacturer’s instructions. Similar but less pronounced results were obtained for pOat3, whereas no signals were detected for Mrps in porcine tissue and cells (data not shown).

### Expression of selected transporter mRNAs with respect to time and cell density

**Figure 8:**
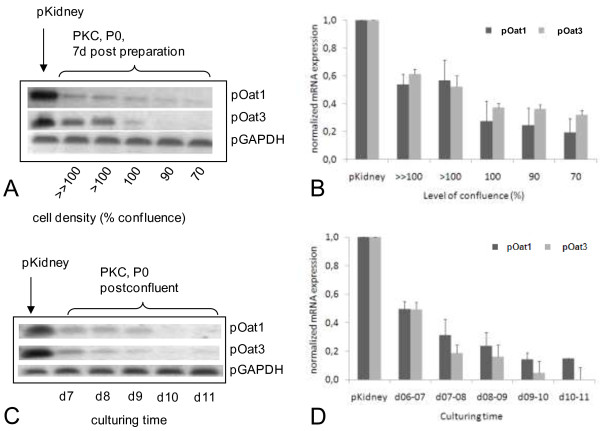
**Effect of level of confluence and culturing time of PKC on pOat mRNA expression.** Color-inverted pictures from representative agarose gels of analyzed samples from cell cultures with varying cell density **(A)** or with varying times in culture **(C)**; summarized densitometric results from cell cultures with varying cell density **(B)** or with varying times in culture **(D)**; data were normalized with the respective pGAPDH value and are expressed as means with SEM from 3-4 **(B)** or 4 **(D)** independent experimentations vs. positive control (pKidney, RNA from porcine renal cortex); level of confluence: 100% represent confluent cultures, values larger than 100% represent postconfluent culture states.

Cells from the same batch of freshly isolated cells (passage 0) were seeded at different cell densities resulting in two different experimental designs. At first, pOat1 and pOat3 expression was tested at various levels of confluence after 7 days in culture. Expression levels decreased for both transporters with decreasing cell density (Figure 8A-B). Then, transporter expression was investigated at the same level of confluence (i.e. at post-confluence) in PKC after different times in culture. Expression levels for both transporters decreased with culturing time (Figure 8C-D).

### Expression of selected transporter on mRNA and protein level in PKC cultures

Porcine Oat1/3 expression was examined over several passages of PKC in culture. At the transcription level, the expression of both transporters decreased in freshly isolated cells (passage 0) during culturing and was absent after passaging and re-culturing of the cells (passage 1 ff.; Table 4). In enriched fractions isolated from confluent PKC cultures (passage 0-3) and from porcine renal cortex tissue pOat1 protein was detectable via Western Blotting analysis (Figure 7) and no change in expression was observed (Table 5). Compared to pOAT1, a lower protein signal was observed for pOat3 in freshly isolated cells and in cultures of passage 0 and 1. Thereafter, pOat3 protein was no longer detectable (Table 5). No specific mRNA or protein was detectable in LLC-PK1 and NRK-52E cells.

**Table 4:** Summarized results of pOat mRNA analysis

**Samples**	**N**	**pOat1**	**pOat3**	**GAPDH**
Porcine kidney (male)	2	+	+	+
PKCm, P0, freshly prepared	4	+	+	+
PKCm, P0, 9-10d	4	+/-	+/-	+
PKCm, P1, 18-24d	4	-	-	+
LLC-PK1	4	-	-	+
NRK-52E	4	-	-	+

N, Number of independent samples analyzed; P, Passage number of cell cultures; d, Total time of culture in days; +, Positive detection, +/- Ambiguous results; -, No detection.

**Table 5:** Summary of pOat protein analysis

**Samples**		**N**	**pOat1**	**pOat3**
rKidney	Cortex	4	+++	++
pKidney	Cortex	4	++	++
PKC	P0, freshly isolated	4	+	+/-
	P0, postconfluent	4	++	+
	P1, postconfluent	4	++	+
	P2, postconfluent	4	++	-
	P3, postconfluent	4	++	-
LLC-PK1	postconfluent	4	-	-
NRK-52E	postconfluent	4	-	-

N, Number of independent samples analyzed; P, Passage number of cell cultures; +, Positive detection of different intensity, +/- Ambiguous results; -, No detection.

### Transporter induction

**Figure 9:**
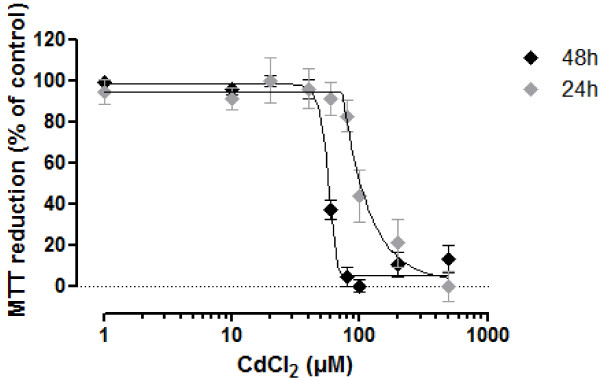
**Cytotoxicity of cadmium chloride in PKC.** Nonlinear fit (5-PL) of normalized and transformed means from 3-4 independent experiments performed in duplicates ± SEM.

To ensure that no cytotoxic side effects could influence the transporter induction experiments, basic cytotoxicity was tested using the MTT reduction assay, which is for many substances one of the most sensitive endpoints for porcine renal cells [8]. The sensitivity of the cells was tested using cadmium chloride as a positive control in a final concentration range of 1-500 μM resulting in a typical sigmoid concentration-response curve (Figure 9). Moreover a time-dependent effect was observed with an EC_50_ of 102.8 μM (95% CI 80.7-131.0 μM, R^2^ = 0.82) and 57.2 μM (95% CI 53.7-60.9 μM, R^2^ = 0.97) for 24 and 48 hours of exposure, respectively. Exposure >48 hours did not result in further increased cytotoxicity (data not shown). The potential transporter inducers (Table 2) did not show any cytotoxic effect after exposure of up to 96 hours, therefore it is very unlikely that they would cause cytotoxicity within the time frame of the induction experiments.

Preliminary experimentation included a comparison of the standard medium (DMEM/F12, with 10% FBS and antibiotics) and a serum-free DMEM/F12, supplemented with transferrin, insulin, hydrocortisone, EGF, triiodothyronine, epinephrine (SingleQuot, Cambrex Bio Science, Verviers, Belgium) and 50 μg/L PGE_1_ (Acros Organics, Geel, Belgium), 5 μg/L selenious acid and antibiotics. No differences in transporter expression were observed (data not shown).

No expression of either Oat1 or Oat3 could be induced at all conditions applied and tested via RT-PCR.

### Kinetics and function of organic anion transport in PKC

Preliminary cytotoxicity testing using the MTT reduction assay as described above confirmed that the chosen substances (transporter substrates and inhibitors) and respective concentrations are non-toxic within 24 hours of exposure (Table 1). Therefore it can be assumed that these substances are unlikely to cause cytotoxicity in the time frame of up to two hours within the transport experiments.

[^14^C]PAH and [^3^H]MTX were chosen as model transport substrates for experimentation. Whereas [^3^H]MTX uptake was negligible, [^14^C]PAH uptake occurred across the basolateral (BLM) and across the brushborder membrane (BBM). Kinetics of BLM uptake of PAH in confluent PKC was measured as a model substrate for pOat1 and pOat3. Uptake showed saturation kinetics (Figure 2A) and non-linear regression analysis yielded a reasonably good fit (R^2^ = 0.85). Similarly, PAH uptake across the BBM, for which the carrier(s) has (have) not been identified, was also characterized by saturable uptake with good fit (R^2^ = 0.92) (Figure 2A).

BBM and BLM uptake was found to be different in capacity and affinity, with BBM uptake being about 3 times higher compared to BLM uptake (Figure 2A). This is also reflected by the K_m_ values of 58.8 ± 12.6 μM and 187.2 ± 70.2 μM (mean ± SEM, n = 3) for uptake across BBM and BLM, respectively. PAH uptake was time-dependent with saturation reached within approximately 5-10 minutes (Figure 2B).

To provide additional validation of the transporter function in PKC, inhibition of PAH and uptake was assessed (Figure 3). MTX, OTA and BSP reduced the PAH BBM uptake by up to 30% (Figure 3A), whereas CIM, FA and OTB showed similar inhibitory effects on the PAH BLM uptake (Figure 3B). All other substances tested (Table 1) were not able to inhibit PAH uptake from either direction.

## Discussion

A good *in vitro* model should approximate an *in vivo*-like behavior as closely as possible in order to reflect most likely the *in vivo* situation [12]. Regarding renal physiology of different species, humans are more closely related to pigs than to rodents [5,13]. Therefore porcine renal cells could prove to be a valuable tool for renal *in vitro* toxicology. Such a renal *in vitro* model should display specific characteristics (e.g. structural organization) and functions (e.g. transepithelial transport) of the respective part of the kidney. This study thus aimed to provide data in order to enable an informed assessment of the suitability of primary porcine renal cells as a model for *in vitro* toxicological testing.

The kidney plays an important role in the elimination of harmful compounds from the body and the reabsorption of vital substances from the glomerular filtrate back into the body. Both types of compounds need to be transported across the membranes of the tubular cells which is realized by specifically localized transporters especially those of the ABC and SLC families [14-25]. A good *in vitro* kidney model must therefore functionally express these transporters in the correct location. In the present study, PKCs were characterized with respect to morphological features (monolayer with the typical epithelial appearance and dome formation). A basolateral-apical polarity can be assumed due to the transporting capabilities observed (see below). This is in accordance with the data from other researchers who additionally tested similarly prepared and cultured PKCs for membrane-specific localization of pMdr1 (abcb1, apical) and pNbc1 (slc4a4, basolateral) using confocal microscopy [26]. These data corroborate earlier findings in PKC, including stable cytokeratin expression patterns up to passage 2 and correspondingly very low but stable vimentin expression as markers of epithelial and mesenchymal cell types, respectively [8,27]. Growth patterns (cellular doubling time, total cell number, monolayer formation) of PKC were found to be similar over three passages although signs of senescence (and/or degeneration) were increasing with time in culture and were apparent especially for cells of passage three. Enlarged cell bodies and cytoskeletal changes are typical phenomena known for senescent cells. Cellular degeneration, especially with respect to change of phenotype to a more fibroblastoid type would possibly be reversible and would include for example a change in marker protein expression. The latter was not observed with cytokeratin and vimentin in earlier studies [8,27].

The data presented here demonstrate that PKC express various transporters at the mRNA level including pMrp1 (abcc1), pMrp2 (abcc2), pOat1 (slc22a6) and pOat3 (slc22a8), whereas pMdr1 (abcb1) and pOatp1a2 (slco1a2) mRNA could not be detected in either the PKCs or in the porcine cortical tissue. The latter might suggest the absence or very low abundance of these transporters in porcine kidney and PKC, a result of the detection technique employed, or a combination of both, i.e. low abundance and limited detection. Results published by Schlatter *et al.* confirm the latter interpretation as they demonstrated pOatp1a2 mRNA in their PKC, but only in freshly isolated cells and with a very faint band after a very high number of PCR cycles [26]. Moreover, they were able to detect pMdr1 mRNA in cultures from different cell preparations using quantitative PCR [26].

To confirm the presence of various transporters in PKC detected at the mRNA level Oat protein expression was assessed also via Western blotting analysis. Due to the fact that transporters are low abundant proteins relative to total cellular protein [28], it was necessary to use higher film exposure times, thereby resulting in relatively high backgrounds. These experiments however confirmed the mRNA expression data for pOat1 and pOat3 in PKC, although the protein bands were different from those obtained with the renal tissue samples from rat and porcine origin. Specific immunoreactive proteins at approximately 75, 45 and 40 kDa were detected. It is suggested, that the large protein (~75 kDa) represents the mature, fully glycosylated form. The smaller proteins, which predominate *in vitro*, may be deglycosylated forms of splice variants [29] and/or forms that are more affected by the experimental procedure, as previously been reported for human OAT1 [30]. However despite that Schlatter *et al.*[26] demonstrated the expression of pMdr1 (abcb1), pMrp1 (abcc1) and pMrp2 (abcc2) in PKC by immunocytochemistry using human protein derived antibodies, Mrp proteins were not detectable in porcine cortical tissue and cells in the experimental set-up reported here. The latter lack of detection, which stands in contrast to the mRNA data, most likely is due to the use of the Mrp antibodies employed. Here, polyclonal anti-human MRP1/2 antibodies were used, that had been predicted to cross-react with the porcine homologues. Obviously this was not the case as these transporters were not detectable in PKC as well as in porcine cortical tissue, although the general function of the antibodies was confirmed by earlier experiments using human tissue (data not shown). These negative results stand also in contrast to data from other investigators showing that Mrps are highly expressed in the kidney of several other species, as reviewed by Klaassen and Aleksunes [24].

In contrast to PKC, the continuous cell lines LLC-PK1 and NRK-52E were negative for all transporters tested at mRNA and protein level. Whereas other investigators described low but detectable functional expression of these transporters in NRK-52E cells [31-34], LLC-PK1 cells were extensively used as models for transporter over-expression by transfection due to their lack of endogenous transporter expression [35-41]. The negative results for NRK-52E cells here are most likely a result of using different strains of NRK-52E cells (ATCC vs. DSMZ), but different cell culture or detection conditions might be causative as well.

Transporter functionality was assessed using radiolabelled PAH and MTX as model transport substrates. PAH uptake occurred across the BLM and BBM, but was found to be different in capacity and affinity, with BBM uptake being about 3 times higher compared to BLM uptake. PAH uptake was time-dependent with saturation reached within approximately 5-10 minutes. MTX, OTA and BSP reduced the PAH BBM uptake by up to 30%, whereas CIM, FA and OTB showed similar inhibitory effects on the basolateral PAH uptake. All other substances tested did not inhibit PAH uptake from either the basolateral or luminal direction.

The observed PAH transport kinetics in PKCs as determined appeared typical for the expected specific transport and thus was considered, at least in part, as attributable to specific expression of pOat1 and pOat3. However, due to the lack of corresponding published data for porcine cells the latter findings could not be corroborated. Indeed, Schlatter and co-workers who also investigated transport in PKC did not report K_m_ values or other parameters that might be suitable for comparison [26]. On the one hand, the calculated K_m_ values for PAH uptake across BBM and BLM were comparable to those observed in primary human proximal tubular cells (BLM K_m_ of 57.5 ± 4.1 μM; BBM K_m_ = 36.6 ± 1.2 μM [28], which is consistent with the high protein homology between porcine and human OATs. The amino acid sequence of pOat1 and pOat3 showed 89% and 81% homology to the human counterparts, respectively [29,42]. Hagos and co-workers investigated two slice variants of pOat1 and one variant of pOat3 expressed in *Xenopus laevis* oocytes [29,42]. While one pOat1 variant did not show any affinity for [^3^H]PAH, the other presented with an apparent K_m_ for [^3^H]PAH of 3.75 ± 1.6 μM. The latter uptake could be inhibited by 0.5 mM glutarate or 1 mM probenecid [25,29], which is comparable to that observed with hOAT1 (K_m_ = 3.9-22 μM) [25]. The reported K_m_ values refer to single transporter proteins overexpressed in oocytes, whereas the presented K_m_ values in our study cannot be assigned to a single transporter but rather to mixed action of at least two different pOats. Nevertheless, based on expression data the observed uptake of PAH is most likely related to pOat1 and to a lesser extent to pOat3. ES, a model substrate for pOat3, was not able to inhibit PAH uptake in our study, which further suggests a predominantly pOat1-mediated uptake. Functional investigations of pOat3 expressed in *X. laevis* oocytes resulted in a high affinity [^3^H]ES transport with an apparent K_m_ of 7.8 ± 1.3 μM with an inhibitory effect of glutarate, DHEAS and probenecid [42], which is similar to hOAT3 (K_m_ = 3.1-9.5 μM) [25]. In our study, probenecid did not show any inhibitory effect on PAH uptake from either the basolateral or the luminal side suggesting that either an additional transporter may be involved in PAH uptake which is not sensitive to probenecid or that the detected transporters pOat1 and pOat3 are indeed slightly different (glycolysation-) variants when compared to their *in vivo* counterparts.

MTX has been shown to interact with several human transporters including hOAT1 (K_m_ = 554-724 μM), hOAT3 (K_m_ = 10.9-21.1 μM), hOAT4 (K_m_ = 17.8 μM), hMRP2 (K_m_ = 250-480; 2,500-3,000 μM) and hMRP4 (K_m_ = 220-1,300 μM) [43]. Cellular MTX uptake was shown to be mediated by hOAT1/3, whereas hOAT4 and hMRPs seem to be involved in MTX efflux [43]. In human kidney cells (HKC), comparable to the PKC cells employed here, hOAT1-associated MTX transport was reported across the BLM with K_m_ = 28.5 ± 1.1 and across the BBM with K_m_ = 80.4 ± 3.4 μM [28]. Based on human data, it can be assumed that MTX uptake is of high affinity/ low capacity, whereas the opposite seems to be true for MTX efflux. If the same holds true for the renal porcine transporters tested here, the results obtained with PKC could be explained by a highly efficient MTX excretion surmounting the concurrent uptake and thus resulting in the low intracellular concentrations determined. Indeed, MTX has been used by others in PKC as a model substrate for Mrp1-6 (abcc1-6), and hypothesized to pump MTX out of the cells [26]. Beyond the latter, MTX may also not be a substrate or a low affinity/low capacity substrate for the transporter variants expressed in PKC. Due to the low intracellular concentrations determined, the question may also be raised whether the transporter variants expressed are truly functional.

mRNA expression levels of pOat 1 and pOat3 in PKCs of passage 0 at different states (cell density and time in culture) was examined and showed that the expression levels for both transporters decreased over time. PKCs of passage 1 did not show any mRNA expression of pOat1 and pOat3 at all. This confirms earlier results from Schlatter and co-workers who observed a down-regulation of pOat1 in culture and detected pOat3 in freshly isolated cells only [26]. The latter observations were also true for other transporters such as pOct1 (slc22a1), which was detectable in freshly isolated cells only, pMrp1 (abcc1) was up-regulated and pMrp2 (abcc2) mRNA expression was down-regulated in culture [26]. To provide a more comprehensive picture of the situation, pOat1/3 expression in PKC was also investigated at the protein level. In contrast to the mRNA data, pOat1 was detectable in PKC up to passage three, whereas pOat3 was present only up to passage one and then at a much lower amount than pOat1. The latter observations suggest that in PKC protein stabilization and/or degradation mechanisms could be altered. This effect and the observed alterations in mRNA expression further confirm the assumption of increasing senescence in the PKC cell strain.

Differences in transporter function amongst individuals may be the result of an up-or down-regulation of OAT expression subsequent to prior exposure to drugs and xenobiotics, or to differences in hormonal status [25]. In addition to the initial expression, post-translational mechanisms including glycosylation, phosphorylation, ubiquitination and SUMOylation seem to be involved in regulating the level and functionality of the expressed transporters [24,25]. While glycosylation appears to be critical in membrane targeting/ trafficking, protein folding and possibly regulation of OAT function, data suggest that phosphorylation and interactions of the OATs with protein partners may change OAT function [24,25]. Indeed T3, DHEAS, TEST and DEX were proposed by several authors [26,44,45] as potential inducers of transporter expression. Accordingly PKC were treated with T3, DHEAS, TEST, BSP, PAH, TCA and DEX and tested whether this would result in a more stable expression of transporters. However, none of the tested substances showed any stimulatory effect on transporter mRNA expression in the tested concentration and time frame. Whether this is due to species differences or to the lacking tissue structure compared to renal slices or tubules used in other studies [44-46] remains to be elucidated.

## Conclusions

A porcine renal *in vitro* model would be cheap, easy to establish, to handle and readily available. The PKC tested here were characterized comprehensively with respect to general features and transport functions. On the basis of the results obtained and the comparison of parts of the data with previously published information, it can be concluded that primary PKC, but not necessarily the resulting PKC cell strain, could represent a valuable model for *in vitro* toxicology that possibly could be used as an alternative to human primary cells, provided a more in-depth characterization of transport and xenobiotic metabolism and toxicity is carried out. Furthermore and pending more in-depth understanding of the expression and functionality of transporters, primary PKC can be used to study toxicokinetics and toxicodynamics of substances as well as tubular toxicity requiring specific transport mechanisms.

## Abbreviations

FBS: Fetal bovine serum; MTT: 3-(4,5-Dimethylthiazol-2-yl)-2,5-diphenyltetrazolium bromide; MDR: Multiple drug resistance protein; MRP: Multidrug resistance-associated protein; OATP: Organic anion transporting polypeptide; SLC: Solute-linked carrier; ABC: ATP-binding cassette transporters; CIM: Cimetidine; ES: Estronesulfate; PAH: p-Aminohippuric acid; MTX: Methotrexate; PROB: Probenecid; FA: Folic acid; BSP: Bromosulphophthalein; OTA: Ochratoxin A; OTB: Ochratoxin B; TCA: Taurocholic acid; T3: Triiodothyronine; DHEAS: Dehydroepi-androsterone sulfate; TEST: Testosterone; DEX: Dexamethasone; PET: Polyethylene terephthalate; SGLT: Sodium-glucose linked transporter or sodium/glucose cotransporter; DAAO: D-amino acid oxidase.

## Competing interests

The authors declare that there are no competing interests.

## Authors’ contributions

AHH designed the study, performed all assays, interpreted the findings and drafted the manuscript. DRD conceived the study and helped to draft the manuscript. Both authors read and approved the final manuscript.

## Supplementary Material

Additional file 1: Figure S1 Amino acid sequence similarities between human, pig, rat and mouse transporter proteins. Click here for file

Additional file 2: Table S1 Comparison of amino acid sequences of transporters. Click here for file

Additional file 3: Table S2 Characteristic transport parameters of selected renal transporters. Click here for file
