# Supplementary material for: Primary porcine proximal tubular cells as an alternative to human primary renal cells in vitro: an initial characterization
Source: BMC Cell Biol. 2013 Dec 5;14:55. doi: 10.1186/1471-2121-14-55 (PMC4234457; doi:10.1186/1471-2121-14-55)
Supplement: Additional file 1: Figure S1 — Amino acid sequence similarities between human, pig, rat and mouse transporter proteins. [file 1471-2121-14-55-S1.docx]

**Supplementary data: Comparison of amino acid sequences of selected transporters**

**Figure S1**: Amino acid sequence similarities between human, pig, rat and mouse transporter proteins


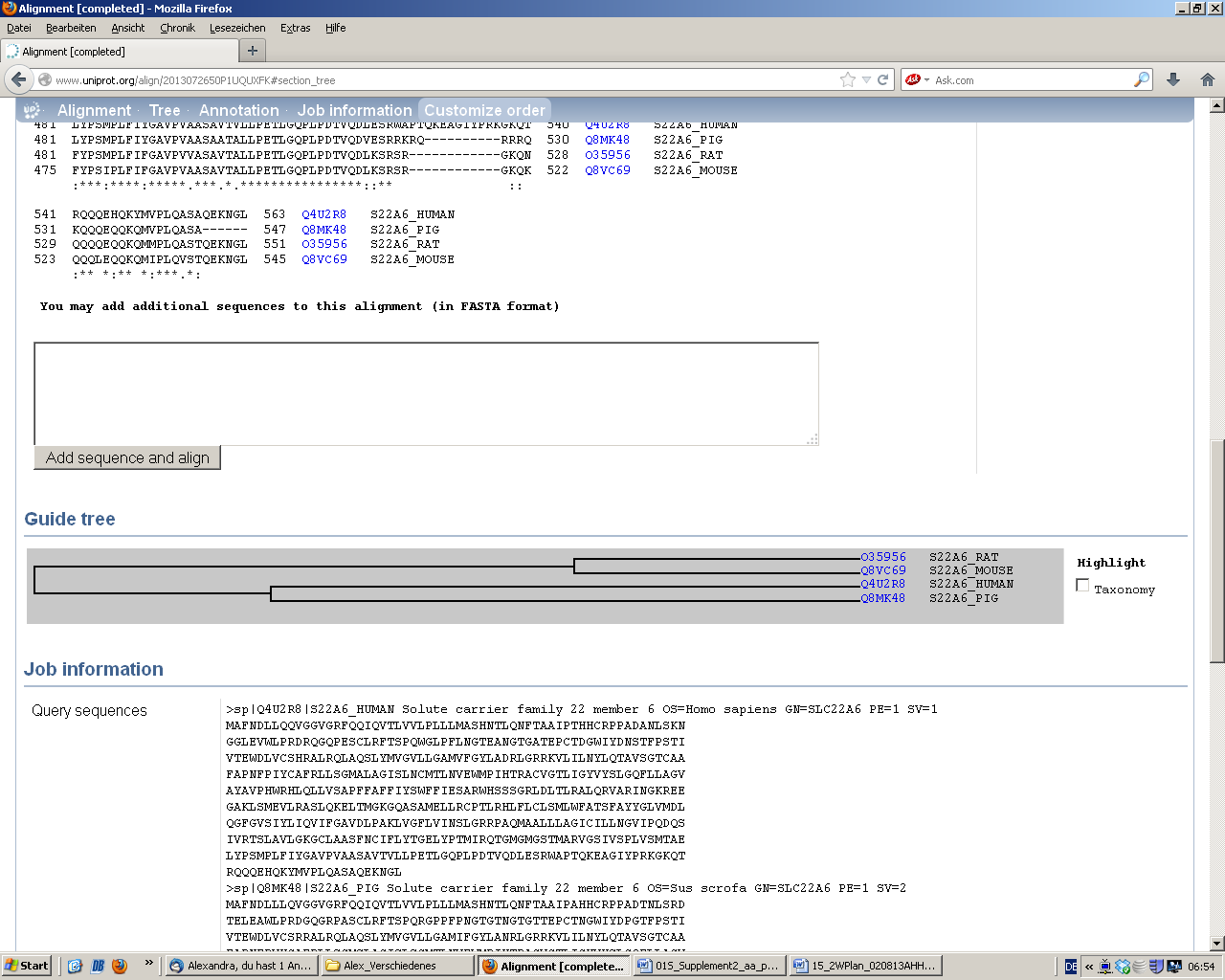


OAT1:


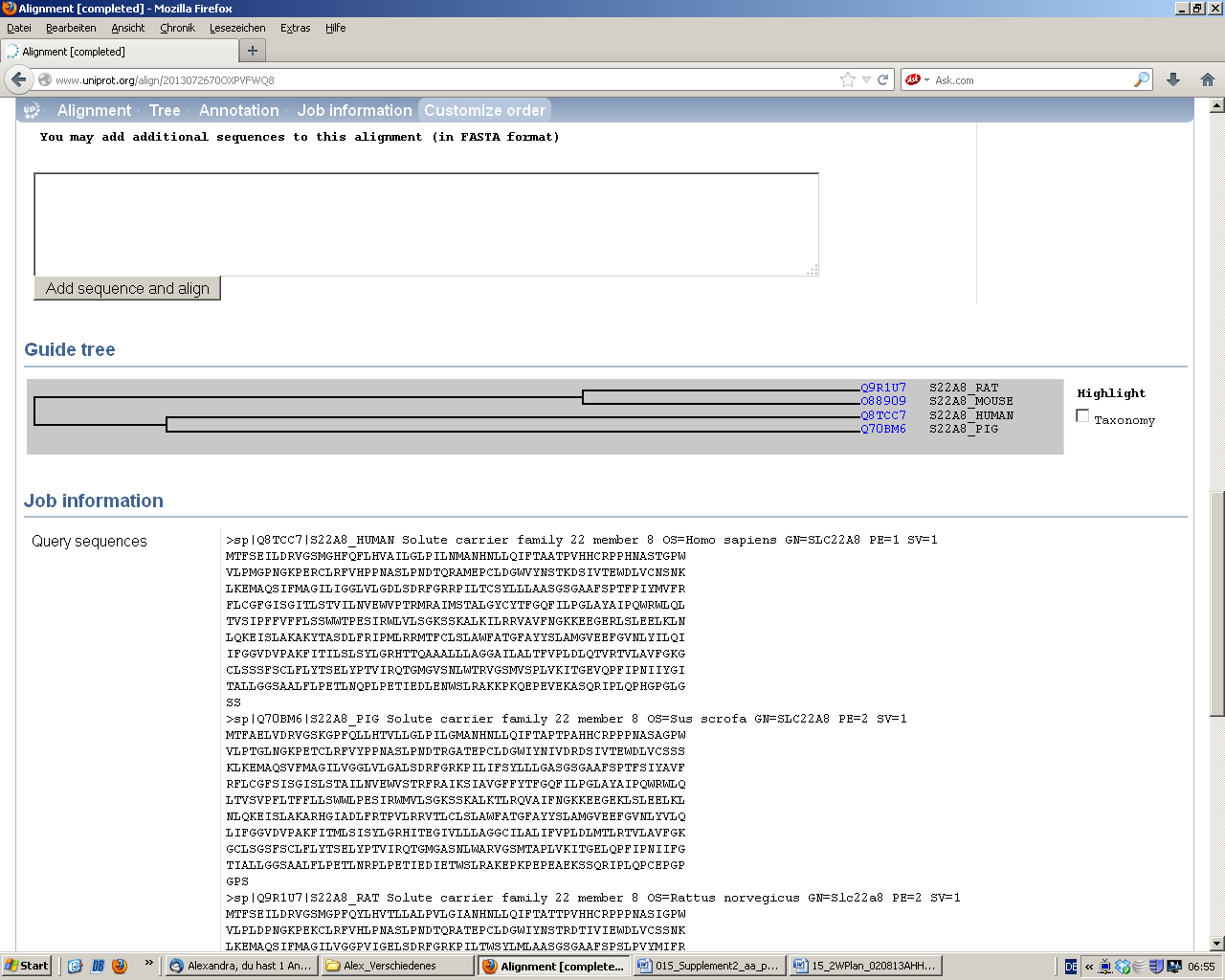


OAT3:


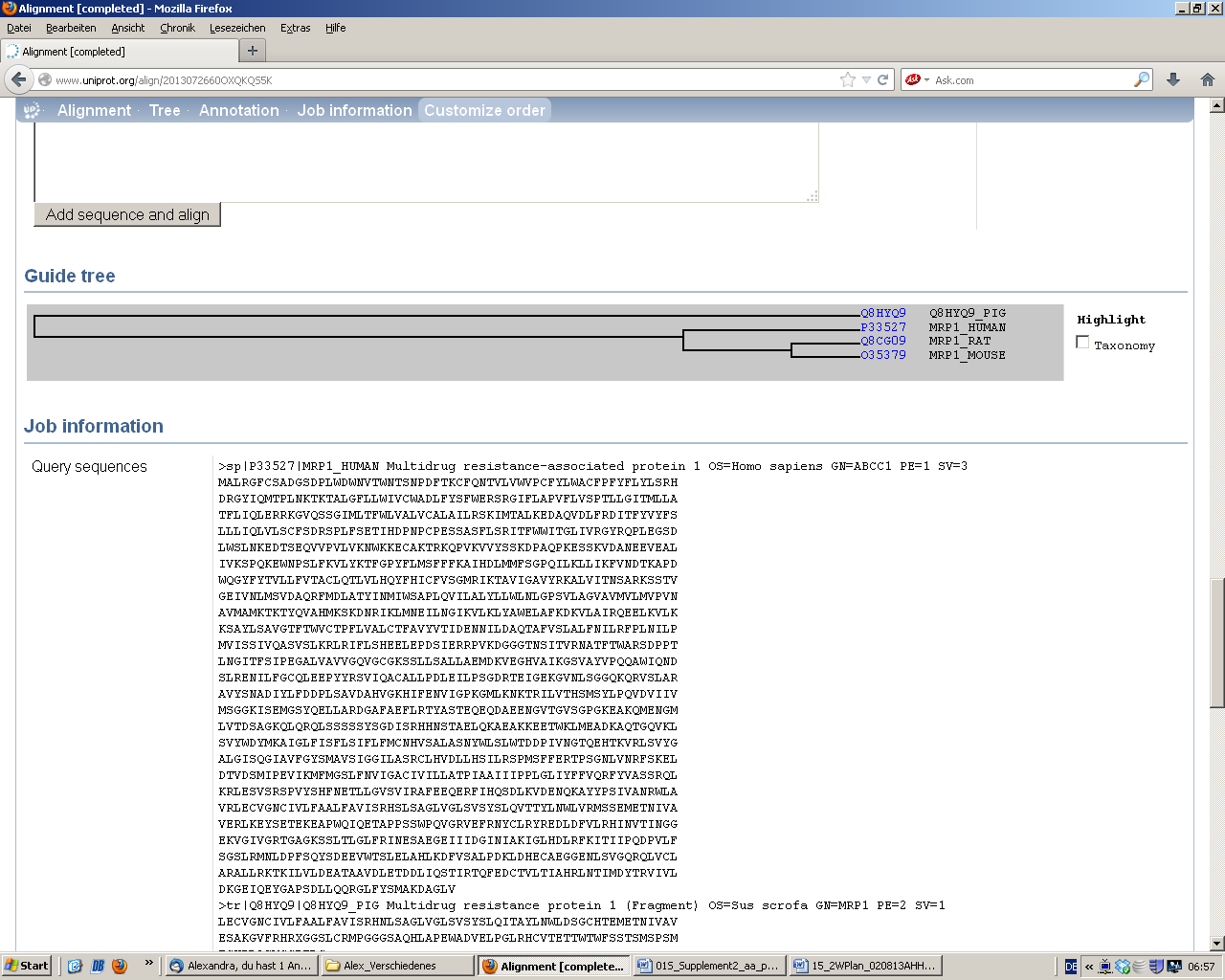


MRP1^a^:


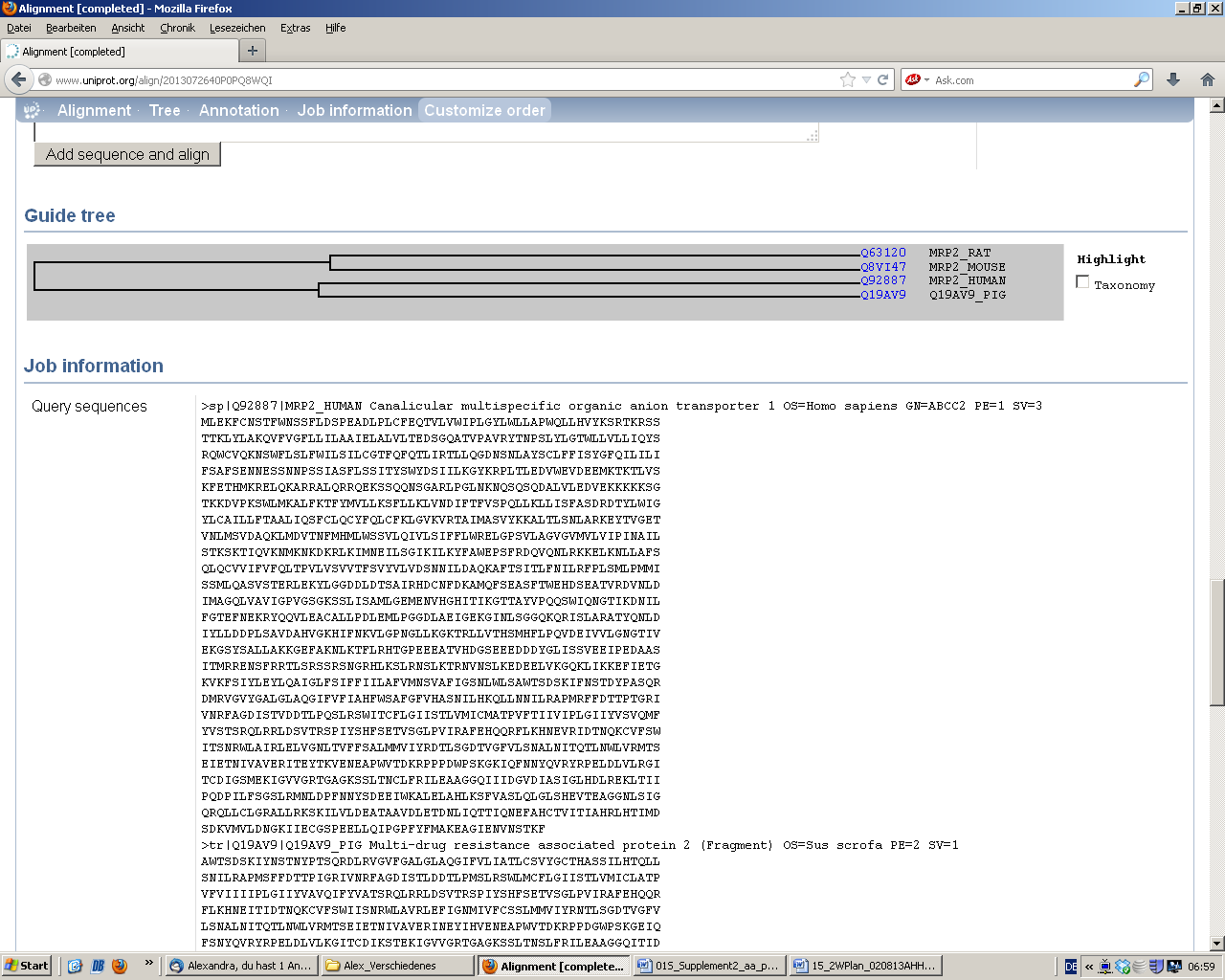


MRP2^a^:


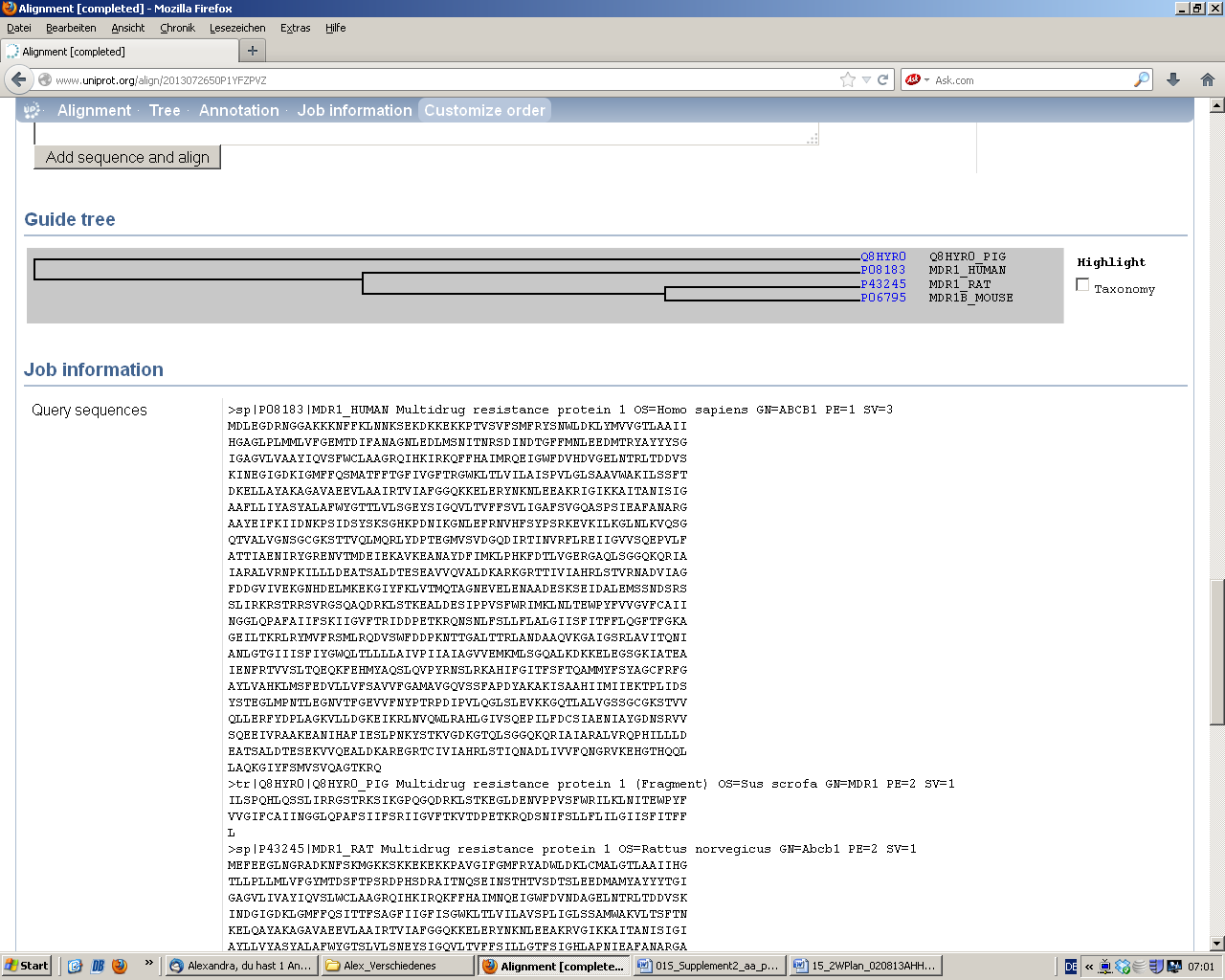


MDR1^a^:

Alignments were performed using UniProt ([www.uniprot.org](http://www.uniprot.org)). ^a^ It is important to note, that these alignments are performed with incomplete sequence data for porcine proteins (see Table S1 for details). Based on comparisons with complete data it is assumed that as soon as full-length sequence data will be available the similarity between human and porcine proteins will increase. OATP1A2: no guide tree available due to missing sequences for rat and mouse.
