# Supplementary material for: Primary porcine proximal tubular cells as an alternative to human primary renal cells in vitro: an initial characterization
Source: BMC Cell Biol. 2013 Dec 5;14:55. doi: 10.1186/1471-2121-14-55 (PMC4234457; doi:10.1186/1471-2121-14-55)
Supplement: Additional file 2: Table S1 — Comparison of amino acid sequences of transporters. [file 1471-2121-14-55-S2.docx]

**Supplementary data: Comparison of amino acid sequences of transporters**

**Table S1: Amino acid sequence similarities (OAT1)**

| Transporter | Protein name | Organism | Length (aa) | Identity (%) | Gene name | UniProt accession number  (date of last update) |
| --- | --- | --- | --- | --- | --- | --- |
| OAT1^a^ | hOAT1-1 (isoform 1) | *Homo sapiens* (Human) | 563 | 100.0 | SLC22A6 | Q4U2R8 (24.07.2013) |
|  | hOAT1-2 (isoform 2) |  | 550 | 97.7 |  |  |
|  | hOAT1-3 (isoform 3) |  | 506 | 89.7 |  |  |
|  | hOAT1-4 (isoform 4) |  | 519 | 92.0 |  |  |
|  | pOat1 | *Sus scrofa* (Pig) | 547 | 86.5 |  | Q8MK48 (03.04.2013) |
|  | rOat1 | *Rattus norvegicus* (Rat) | 551 | 86.0 |  | O35956 (24.07.2013) |
|  | mOat1 | *Mus musculus* (Mouse) | 545 | 83.3 |  | Q8VC69 (03.04.2013) |
| OAT3^a^ | hOAT3-1 (Isoform 1) | *Homo sapiens* (Human) | 542 | 100.0 | SLC22A8 | Q8TCC7 (24.07.2013) |
|  | hOAT3-2 (Isoform 2) |  | 551 | 98.2 |  |  |
|  | hOAT3-3 (Isoform 3) |  | 110 | 12.3 |  |  |
|  | hOAT3-4 (Isoform 4) |  | 451 | 83.2 |  |  |
|  | hOAT3-5 (Isoform 5) |  | 419 | 77.3 |  |  |
|  | pOat3 | *Sus scrofa* (Pig) | 543 | 81.8 |  | Q70BM6 (29.05.2013) |
|  | rOat3 | *Rattus norvegicus* (Rat) | 536 | 78.8 |  | Q9R1U7 (24.07.2013) |
|  | mOat3 | *Mus musculus* (Mouse) | 537 | 77.5 |  | O88909 (24.07.2013) |
| MRP1^a^ | hMRP1-1 (isoform 1) | *Homo sapiens* (Human) | 1,531 | 100.0 | ABCC1 | P33527 (24.07.2013) |
|  | hMRP1-2 (isoform 2) |  | 1,472 | 96.1 |  |  |
|  |  |  |  |  |  |  |
|  | hMRP1-3 (isoform 3) |  | 1,475 | 96.3 |  |  |
|  | hMRP1-4 (isoform 4) |  | 1,466 | 95.8 |  |  |
|  | hMRP1-5 (isoform 5) |  | 1,416 | 92.5 |  |  |
|  | hMRP1-6 (isoform 6) |  | 1,407 | 91.9 |  |  |
|  | hMRP1-7 (isoform 7) |  | 1,410 | 92.1 |  |  |
|  | hMRP1-8 (isoform 8) |  | 1,351 | 88.2 |  |  |
|  | pMrp1 | *Sus scrofa* (Pig) | 134 | n.a.^b^ |  | Q8HYQ9 (fragment sequence) (03.04.2013) |
|  | rMrp1-1 (isoform 1) | *Rattus norvegicus* (Rat) | 1,532 | 87.4 |  | Q8CG09 (24.07.2013) |
|  | rMrp1-2 (isoform 2) |  | 1,523 | 86.9 |  |  |
|  | mMrp1 | *Mus musculus* (Mouse) | 1,528 | 88.1 |  | O35379 (24.07.2013) |
| MRP2^c^ | hMRP2 | *Homo sapiens* (Human) | 1,545 | 100.0 | ABCC2 | Q92887 (24.07.2013) |
|  | pMrp2 | *Sus scrofa* (Pig) | 483 | n.a.^b^ |  | Q19AV9 (fragment sequence) (29.05.2013) |
|  | rMrp2 | *Rattus norvegicus* (Rat) | 1,541 | 77.4 |  | Q63120 (24.07.2013) |
|  | mMrp2 | *Mus musculus* (Mouse) | 1,543 | 77.5 |  | Q8VI47 (24.07.2013) |
| MDR1^c^ | hMDR1 | *Homo sapiens* (Human) | 1,280 | 100.0 | ABCB1 | P08183 (24.07.2013) |
|  | pMdr1 | *Sus scrofa* (Pig) | 121 | n.a.^b^ |  | Q8HYR0 (fragment sequence) (29.05.2013) |
|  | rMdr1 | *Rattus norvegicus* (Rat) | 1,277 | 80.0 |  | P43245 (24.07.2013) |
|  | mMdr1 | *Mus musculus* (Mouse) | 1,276 | 80.0 |  | P06795 (24.07.2013) |
| OATP1A2^a^ | hOATP1A2-1 | *Homo sapiens* (Human) | 670^a^ | 100.0 | **SLCO1A2** | P46721 (24.07.2013) |
|  | hOATP1A2-2 |  | 579^a^ | 81.0 |  |  |
|  | pOatp1a2 | *Sus scrofa* (Pig) | 667^b^ | 83.7 |  | H6WA55 (01.05.2013) |
|  | rOatp1a2 | *Rattus norvegicus* (Rat) | No data | n.a.^b^ |  | None |
|  | mOatp1a2 | *Mus musculus* (Mouse) | No data | n.a.^b^ |  | None |

^a^BLAST was performed using UniProt ([www.uniprot.org](http://www.uniprot.org)) using isoform 1 of the human protein as the ‘canonical’ sequence for comparison; ^b^no complete sequence available for comparison; ^c^BLAST was performed using UniProt ([www.uniprot.org](http://www.uniprot.org)) using the human protein sequence for comparison;
