# Supplementary material for: Primary porcine proximal tubular cells as an alternative to human primary renal cells in vitro: an initial characterization
Source: BMC Cell Biol. 2013 Dec 5;14:55. doi: 10.1186/1471-2121-14-55 (PMC4234457; doi:10.1186/1471-2121-14-55)
Supplement: Additional file 3: Table S2 — Characteristic transport parameters of selected renal transporters. [file 1471-2121-14-55-S3.docx]

**Supplementary data: Characteristic parameters of selected renal transporters**

**Table S2: Characteristic transport parameters of selected renal transporters**

| Protein name  (gene name) | Parameter | Human | Pig | Rat | Mouse |
| --- | --- | --- | --- | --- | --- |
| OAT1  (SLC22A6) | K_M (PAH, µM)_ | 5-9 [[1](#_ENREF_1)] | 3.7 [[1](#_ENREF_1), [2](#_ENREF_2)] | 14.3 [[1](#_ENREF_1), [3](#_ENREF_3)]; 70 [[1](#_ENREF_1), [4](#_ENREF_4)] | 37.3 [[1](#_ENREF_1), [5](#_ENREF_5)] |
|  | Expression | BLM [[6](#_ENREF_6)] | BLM [[7](#_ENREF_7)] | BLM [[3](#_ENREF_3), [4](#_ENREF_4)] | BLM^a^ |
|  | Selected substrates | PAH, CIM, FUR, IND, MTX, OTA [[8](#_ENREF_8)] | PAH, PROB [[2](#_ENREF_2)] | PAH, FUR, IND, MTX, PROB [[3](#_ENREF_3), [4](#_ENREF_4)] | PAH, PROB, IND, MTX [[5](#_ENREF_5)] |
| OAT3  (SLC22A8) | K_M (PAH, µM)_ | 50-100 [[1](#_ENREF_1)] | No data available | 60-65 [[1](#_ENREF_1)] | no data available |
|  | K_M (ES, µM)_ | 3.1-9.5 [[1](#_ENREF_1)] | 7.8 [[1](#_ENREF_1), [9](#_ENREF_9)] | 2.3-5.3 [[1](#_ENREF_1)] | no data available |
|  | Expression | BLM [[10](#_ENREF_10)] | BLM [[9](#_ENREF_9)] | BLM [[11](#_ENREF_11)] | BLM^a^ |
|  | Selected substrates | PAH, CIM, CA, DHEAS, ES, MTX, OTA, TCA [[8](#_ENREF_8)] | DHEAS, PROB [[9](#_ENREF_9)] | PROB, FUR [[11](#_ENREF_11)] | BSP, PRO, TCA, ES [[12](#_ENREF_12)]; CIM [[13](#_ENREF_13)] |

^a^ Inferred from sequence or structural similarity. Source: UniProtKB; For MRP1 (ABCC1), MRP2 (ABCC2), MDR1 (ABCB1) and OATP1A2 (SLCO1A2) there were not enough data available for comparison;

**References**

1. Srimaroeng, C., J. Perry, and J. Pritchard, *Physiology, structure, and regulation of the cloned organic anion transporters.* Xenobiotica, 2008. **38**(7-8): p. 889-935.

2. Hagos, Y., et al., *Cloning of the pig renal organic anion transporter 1 (pOAT1).* Biochimie 2002. **84**(12): p. 1221-1224.

3. Sekine, T., et al., *Expression cloning and characterization of a novel multispecific organic anion transporter.* J Biol Chem, 1997. **272**(30): p. 18526-9.

4. Sweet, D., N. Wolff, and J. Pritchard, *Expression cloning and characterization of ROAT1. The basolateral organic anion transporter in rat kidney.* J Biol Chem, 1997. **272**(48): p. 30088-95.

5. Kuze, K., et al., *Heterologous expression and functional characterization of a mouse renal organic anion transporter in mammalian cells.* J Biol Chem, 1999. **274**(3): p. 1519-24.

6. Hosoyamada, M., et al., *Molecular cloning and functional expression of a multispecific organic anion transporter from human kidney.* Am. J. Physiol., 1999. **276**: p. F122-F128.

7. Sekine, T., H. Miyazaki, and H. Endou, *Molecular physiology of renal organic anion transporters.* American Journal of Physiology 2006. **290**(Renal Physiology): p. F215-F261.

8. Klaassen, C. and L. Aleksunes, *Xenobiotic, bile acid, and cholesterol transporters: function and regulation.* Pharmacol Rev, 2010. **62**(1): p. 1-96.

9. Hagos, Y., et al., *Functional expression of pig renal organic anion transporter 3 (pOAT3).* Biochimie, 2005. **87**(5): p. 421-424.

10. Motohashi, H., et al., *Gene Expression Levels and Immunolocalization of Organic Ion Transporters in the Human Kidney.* J Am Soc Nephrol, 2002. **13**: p. 866-874.

11. Lash, L., et al., *Role of rat organic anion transporter 3 (Oat3) in the renal basolateral transport of glutathione.* Chem Biol Interact., 2007. **170**(2): p. 124-34.

12. Sweet, D., et al., *Impaired organic anion transport in kidney and choroid plexus of organic anion transporter 3 (Oat3 (Slc22a8)) knockout mice.* J Biol Chem, 2002. **277**(30): p. 26934-43.

13. Kobayashi, Y., et al., *Renal transport of organic compounds mediated by mouse organic anion transporter 3 (mOat3): further substrate specificity of mOat3.* Drug Metab Dispos, 2004. **32**(5): p. 479-83.

14. Leier, I., et al., *ATP-dependent para-aminohippurate transport by apical multidrug resistance protein MRP2.* Kidney Int., 2000. **57**(4): p. 1636-42.

15. Smeets, P., et al., *Contribution of multidrug resistance protein 2 (MRP2/ABCC2) to the renal excretion of p-aminohippurate (PAH) and identification of MRP4 (ABCC4) as a novel PAH transporter.* J Am Soc Nephrol., 2004. **15**(11): p. 2828-35.
